# The inverse association between circulatory placental biomarkers in early pregnancy and maternal body mass index

**DOI:** 10.1016/j.placenta.2026.03.004

**Published:** 2026-03-04

**Authors:** Nooria Atta, Cleo Pike, Tabitha Wishlade, Ulla Sovio, Clive Petry, Sam Lockhart, Ken K Ong, Ieuan A Hughes, Sandra F Goodburn, Gordon Smith, Catherine Aiken, Stephen O’Rahilly

**Affiliations:** 1 University of Cambridge, Medical Research Council Metabolic Diseases Unit, Institute of Metabolic Science-Metabolic Research Laboratories, Cambridge, CB2 0QQ, UK; 2 Department of Clinical Biochemistry, Addenbrooke's Hospital, Cambridge, CB2 0QQ, UK; 3 Department of Obstetrics and Gynaecology, University of Cambridge; The Rosie Hospital NIHR Cambridge Biomedical Research Centre, Cambridge, CB2 0QQ, UK; 4 The Loke Centre for Trophoblast Research, Department of Physiology, Development and Neuroscience, University of Cambridge, Cambridge, CB2 0QQ, UK; 5 Queen's University Belfast, School of Medicine, Dentistry and Biomedical Sciences, Wellcome-Wolfson Institute for Experimental Medicine, Belfast BT9 7BL, UK; 6 MRC Epidemiology Unit and NIHR Cambridge Biomedical Research Centre, Wellcome-MRC Institute of Metabolic Science, University of Cambridge, Cambridge, CB2 0QQ, UK; 7 Department of Paediatrics, Addenbrooke's Hospital, University of Cambridge, Cambridge, CB2 0QQ, UK

## Abstract

High maternal body-mass-index (BMI) is linked to adverse pregnancy outcomes, partly through impaired placental development. An inverse association between BMI and placental biomarkers has been described, however it is unclear whether this relationship is restricted to obesity or occurs across the full-BMI range. We assessed the relationship between maternal BMI and placental biomarkers across multiple cohorts.

**Methods:**

We analysed three UK cohorts: ALSPAC (n=994), CBGS (n=1,202) and POPS (n=3,869). Concentrations of growth-differentiation-factor15 (GDF15), beta-human chorionic gonadotropin (βhCG), pregnancy-associated plasma protein-A (PAPP-A), and alpha-fetoprotein (AFP) were measured at 8–16 weeks of gestation in maternal serum and expressed as gestational age–adjusted z-scores. Cohort-specific regression and individual-participant-data-meta-analyses (IPD-MA) were used.

**Results:**

Across cohorts, each 1SD increase in maternal BMI was associated with significant reduction in: GDF15 (β: -0.16, 95%CI: -0.23 to -0.10; p<0.001), βhCG (β: - 0.26, 95%CI: -0.28 to -0.23; p<0.001), PAPP-A (β: -0.38 95%CI: -0.41 to -0.36; p<0.001), and AFP (β: -0.21, 95%CI: -0.24 to -0.18; p<0.001). Associations were linear across the full BMI range, not driven solely by obesity. Biomarker levels correlated more strongly with early-pregnancy BMI than with estimated blood volume and associated directionally with the maternal polygenic-risk-scores (PRS) for BMI.

**Conclusions:**

Maternal BMI across its full range was inversely associated with biomarkers arising from, or transferred through, the placenta. We propose that the maternal caloric environment may regulate the development of the materno-fetal interface and its capacity for nutrient transfer, supporting similar rates of early fetal-growth in the face of substantial inter-individual variation in maternal body mass.

## Introduction

Maternal nutritional status in early pregnancy, particularly caloric status as proxied by body mass index (BMI) [1], has a significant impact on pregnancy outcomes, including fetal growth trajectories [2–4]. There is a U-shaped association between maternal BMI and birth weight, with an excess of both large-for-gestational age and small-for-gestational age infants in women with higher BMI [5–7]. Mechanistic studies in humans to determine the precise effects of maternal adiposity on the earliest stages of placental and fetal development are challenging. Recently however, Schenkelaars et al [8] used 3D ultrasound imaging combined with artificial intelligence to measure uteroplacental vascular volume (UPVV) in early pregnancy. They reported that maternal BMI was associated with a significant reduction in UPVV and suggested that obesity may impair early placental development. Previous studies have reported the relationship between maternal BMI and the circulating maternal concentrations of a range of protein biomarkers either made in by the trophoblast (e.g. βhCG, PAPP-A, GDF15) or synthesized in the fetus and passed through the placenta (e.g. AFP) into maternal blood [9–12]. The findings of such studies have been largely consistent, reporting an inverse correlation between placental biomarkers and maternal BMI. Where an explanation for these relationships has been suggested, it has almost always involved attributing the effect to the fact that larger women have higher blood volumes and that lower levels of biomarkers are the result of haemodilution. Indeed, relationships between measures such as AFP and BMI are so well established that, in clinical practice, body weight adjustment of such markers is often adopted [10,12,13].

In undertaking this study, we asked several questions. Firstly, is the relationship between maternal BMI and pregnancy biomarkers particularly driven by obesity or does it occur across the full range of maternal adiposity, from underweight to obese? Secondly, if such a relationship does exist, how likely is it to be entirely driven by haemodilution? Thirdly, are the relationships with maternal BMI similar when comparing biomarkers that are secreted by the trophoblast (βhCG, PAPP-A, GDF15) or made in the fetus and transported into the maternal blood (AFP)?

The inverse association of maternal BMI with biomarkers of pregnancy may reflect a specific adverse impact of obesity on placental development. In our analyses, we have been careful to examine the associations of BMI across its entire spectrum, reasoning that relationships that persist across the full range of BMIs would suggest different underlying biology.

In order to obtain data sufficiently powered to address these questions, we measured biomarkers in >6,000 participants from three independent prospective cohorts. In the largest cohort, which had been densely genotyped; we also analyzed the relationship between the mother’s polygenic risk score for BMI and placental biomarkers.

## Methods

### Study design

#### Data used in this analysis were obtained from three cohort studies conducted in the UK:

##### Cohort 1

ALSPAC (The Avon Longitudinal Study of Parents and Children, formerly the Avon Longitudinal Study of Pregnancy and Childhood) is a birth cohort in Avon, UK. The cohort initially recruited 14,541 pregnant women with expected dates of delivery between 1st April 1991 and 31st December 1992. Of these pregnancies, 338 were from a woman who had already enrolled with a previous pregnancy, meaning 14,203 unique mothers were initially enrolled in the study. As a result of the additional phases of recruitment, a further 630 women who did not enrol originally have provided data since their child was 7 years of age. This provides a total of 14,833 unique women (G0 mothers) enrolled in ALSPAC as of September 2021. Full details of the study can be found elsewhere [14,15]. The study website (http://www.bristol.ac.uk/alspac/researchers/our-data/) contains details of all the data that is available through a fully searchable data dictionary and variable search tool.

Participants whose serum biomarkers were measured using research samples collected between 8-16 weeks gestation are included in this study. Maternal height and weight were self-reported.

##### Cohort 2

The Cambridge Baby Growth Study (CBGS) recruited women aged ≥16 years, irrespective of parity, in early pregnancy (~12 weeks) at the Rosie Maternity Hospital, Cambridge, UK, between April 2001 - March 2009. Full details are published elsewhere [16]. Maternal characteristics including height and weight were self-reported at recruitment. Biomarkers were measured in blood samples collected during early pregnancy, either as part of routine clinical screening (βhCG and AFP) or as research assays (GDF15 and PAPP-A).

##### Cohort 3

The Pregnancy Outcome Prediction (POP) study is a birth cohort that recruited nulliparous women with singleton pregnancies at The Rosie Hospital, Cambridge, UK, between January 2008 - July 2012. Details of the POPS cohort are published elsewhere [17–19]. Recruitment and initial blood sampling occurred at ~12 weeks gestation. Maternal weight was measured at the booking visit; height was recorded at one of the study visits [20].

For all cohorts, we analysed results from participants with singleton pregnancies who had complete maternal height, weight, and relevant biomarker data available between 8-16 weeks of gestation. In the CBGS and POPS cohorts, gestational age was measured using early-pregnancy dating ultrasound scans and recorded in a week + day format. In ALSPAC, gestational age was estimated based on last menstrual period (LMP) and, where necessary, confirmed by antenatal ultrasound scan [21]. It was recorded as complete weeks of gestation.

### Study variables

Maternal BMI (kg/m^2^), the primary exposure, was calculated from measured or self-reported maternal height and weight. Maternal estimated blood volume (EBV) [22,23] was estimated using the Lemmens-Bernstein-Brodsky (LBB) equation [24]. There are pregnancy-specific methods of estimating BV available, however these are primarily derived for use in the third trimester [25] and were therefore not applied to our early pregnancy data.

Circulating maternal serum levels of βhCG (mIU/l), PAPP-A (IU/ml), AFP (IU/ml), and GDF15 (pg/ml) were obtained (Supplementary Table 1). Circulating biomarker concentrations were log-transformed. They were then converted to gestational age–adjusted z-scores (in days). This approach accounted for rapid changes with advancing gestation and for differences in gestational age within and between cohorts.

### Statistical analysis

Descriptive statistics were used to summarize baseline characteristics for each cohort. Means and standard deviations (SD) were reported for continuous variables and proportions for the categorical variables.

We first examined correlations among the variables included in this study. Since the EBV is a function of BMI (LBB equation = 70/[√BMI÷22]), each exposure was modeled separately.

Linear regression models were used to assess individual associations between the exposures and biomarkers. Effect sizes (β coefficients) and 95% confidence intervals were estimated, along with model performance metrics including R^2^ and Akaike Information Criterion (AIC). Models were adjusted for maternal age (years), ethnicity, and fetal sex. Regression models were evaluated for key assumptions, including linearity, homoscedasticity, and normality of residuals, and influential observations were assessed using standardized residuals and leverage statistics. No imputation was performed for missing data. Cases with missing information for anthropometrics and/or placental biomarkers were assumed to be missing at random, given that the full cohorts had previously undergone analysis.

The covariates were selected a priori based on biological plausibility, known associations with either the exposures or outcomes, and availability of data across the cohorts. Due to the very high proportion of women who identified as white ethnicity (~95% across all cohorts), ethnicity was modelled as white vs non-white in our multivariable models.

For biomarkers measured in more than one cohort, we pooled the raw data from each cohort to jointly estimate associations while accounting for cohort-specific variability using random effects models in individual participant data meta-analyses (IPD-MA).

To illustrate trends across the BMI spectrum, maternal BMI was categorized into quintiles and mean (SD) biomarker concentrations were compared across the quintiles using one-way ANOVA with post-hoc t-tests and Bonferroni correction.

Polygenic risk scores (PRS) for BMI were generated using data from the POPS cohort. DNA from maternal blood was genotyped using the Illumina Infinium Global Screening Array Kit (GSA v3), followed by stringent quality control and kinship filtering prior to imputation. We applied a BMI polygenic risk score including nearly 1.3 million variants [26] from the PGS Catalog [27] to 3420 of POPS participants. The genotyping and imputation methods used in POPS are given in detail elsewhere [28]. To calculate the BMI risk scores within POPS we used the PGS Catalog polygenic score calculator pipeline [29]. To control for population stratification, we calculated the first 10 principal components (PCs) of ancestry using plink2 on typed variants with a minor allele frequency >0.05 that had been pruned for linkage disequilibrium using the indep-pairwise function (window size of 50, step size of 5, r^2^ threshold of 0.1).

Individual PRS values were standardized within the study population and incorporated into regression models as predictors alongside measured maternal BMI. To quantify the variance in BMI not explained by the PRS, residual BMI values were derived by regressing observed BMI on the PRS and using the residuals in subsequent analyses. Regression models evaluating the association of PRS and residual BMI with placental biomarkers were adjusted for maternal age, PC1–PC3, and fetal sex, whereas models relating observed BMI (phenotype) to placental biomarkers were adjusted for maternal age, ethnicity, and fetal sex.

All statistical analyses were conducted using R version 4.3.3 (R Core Team, Vienna, Austria). Two tailed tests were applied, and statistical significance was set at *p* <0.05.

We used data from three ethically approved cohorts. Ethical approval for ALSPAC was obtained from the ALSPAC Ethics and Law Committee and the Local Research Ethics Committees. Informed consent for the use of all data collected was obtained from participants following the recommendations of the ALSPAC Ethics and Law Committee at the time. Participants can contact the study team at any time to retrospectively withdraw consent for their data to be used. Study participation is voluntary and during all data collection sweeps, information was provided on the intended use of data [14,15].

The CBG study was approved by the Cambridge Research Ethics Committee (LREC 00/325) [16]. The POPS study was approved by the Cambridgeshire 2 Research Ethics Committee (07/H0308/163), with additional approval for use of routinely collected data from the South Central (Berkshire) (12/SC/0344) [17,20]. All participants provided written informed consent. Access to the datasets was granted upon review and approval of the submitted analysis plan (Supplementary Table 2).

## Results

### (i) Baseline characteristics

A total of 6,065 participants from three prospective cohorts were included (ALSPAC; n=994, CBGS; n=1,202, and POPS; n=3,869). These cohorts represent 3 different decades, with ALPAC recruiting in the early 1990s, CBGS in the early 2000s, and POPS early 2010s.

Across the cohorts, there were significant differences in maternal age; with ALSPAC participants being significantly younger than those in CBGS and POPS (Table 1). Most women (~95%) reported their ethnicity as White in all 3 cohorts (Table 1).

There were differences in maternal height across cohorts; these reached statistical significance, but with mean values spanning <1.5cm are unlikely to be of clinical relevance. Maternal weight was also different between cohorts, being lowest in ALSPAC (61.4±10.4 kg) and highest in POPS (68.4±13.4 kg). Correspondingly, the lowest mean BMI was observed in ALSPAC (22.7±3.6 kg/m2), compared to CBGS (24.1±4.6 kg/m2), and POPS (25.1±4.7 kg/m2; Table 1). The majority (63.5%) of women included in the study overall were within the BMI <25 kg/m2 category (n=3447), with 25% (n=1359) in the BMI 25-29.9 kg/m2 range, 10.4% (n=565) in the BMI 30-40 kg/m2 range, and 1.1% (n=57) had BMI >40 kg/m2. Mean (SD) biomarker levels measured at early pregnancy are shown in Table 2.

### (ii) Association between maternal BMI and placental biomarkers

**Figure 1:**
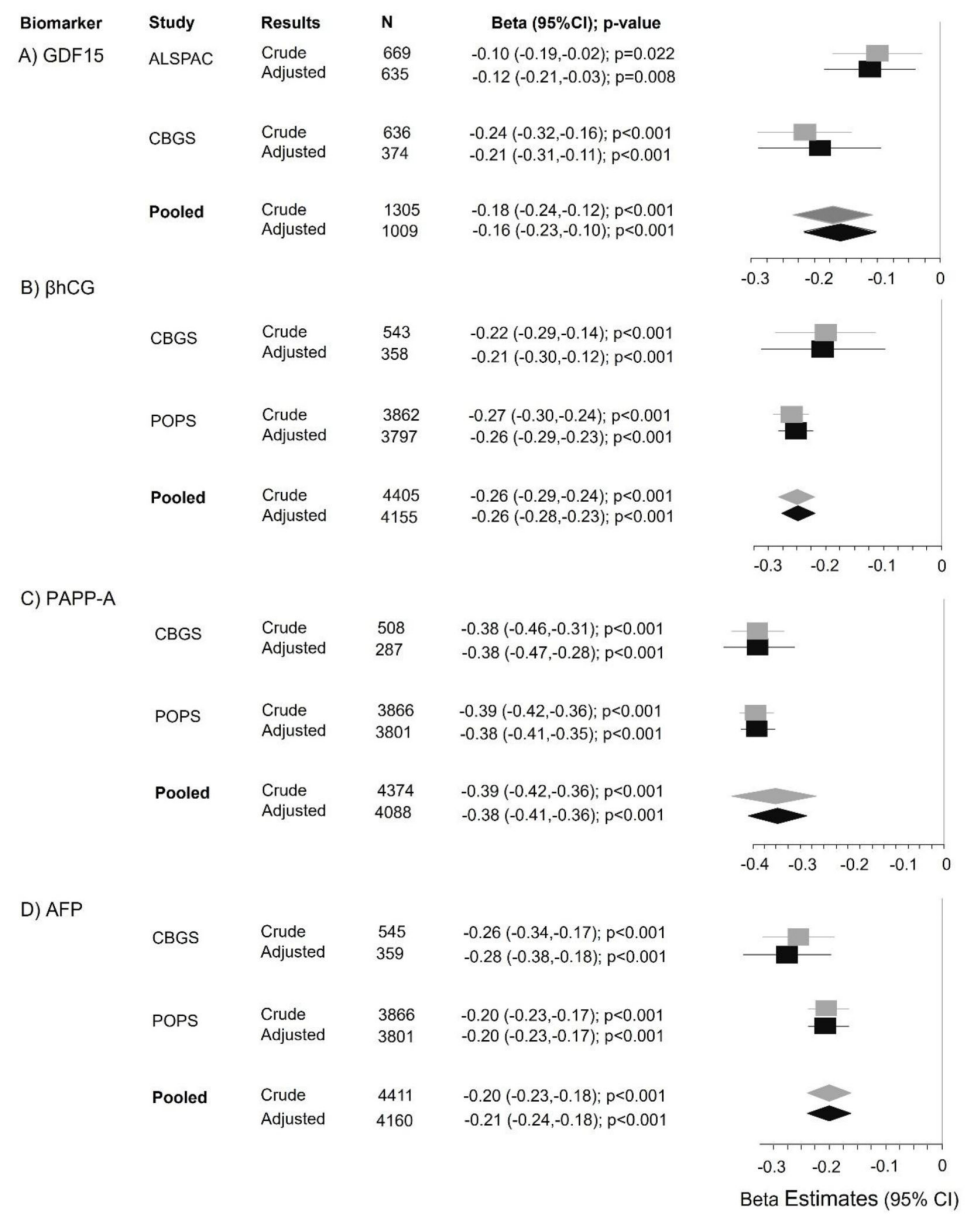
Results from individual participant data meta-analysis (IPD-MA); β coefficients are expressed per 1-SD increase in the BMI. The forest plots show cohort-specific and pooled associations between maternal BMI and placental biomarkers. GDF15: growth differentiation factor15; βhCG: beta-human chorionic gonadotropin; PAPP-A: pregnancy associated plasma protein A; AFP: alpha-fetoprotein. *Adjustments: maternal age (standardized), ethnicity and fetal sex.

In both the ALSPAC and CBGS cohorts, there was an inverse association between maternal BMI and serum concentration of GDF15 (Fig 1). These associations persisted after adjustment for relevant co-variates (maternal age, ethnicity, and fetal sex). In the combined cohort analysis, a 1-SD higher BMI was associated with a lower gestational age-adjusted z-score for GDF15 (β: -0.16, 95%CI: -0.23 to -0.10; p<0.001).

In both the POPS and CBGS cohorts, maternal BMI was inversely associated with βhCG (Fig 1). These associations persisted after adjustment for relevant co-variates. In combined cohort analysis, a 1-SD higher BMI was associated with a significantly lower gestational age-adjusted z-score for βhCG (β: -0.26, 95%CI: -0.28 to -0.23; p<0.001).

Similarly, maternal BMI was inversely associated with PAPP-A in both the POPS and CBGS cohorts (Fig 1). In the combined cohort, a 1-SD higher BMI was associated with a lower gestational age–adjusted PAPP-A z-score (β: -0.38, 95%CI: -0.41 to -0.36; p<0.001).

Maternal BMI was inversely associated with AFP in both the POPS and CBGS cohorts (Fig 1). These associations persisted after adjustment for the co-variates. In the combined cohort, a 1-SD higher BMI was associated with a lower gestational age–adjusted AFP z-score (β: -0.21, 95%CI: -0.24 to -0.18; p<0.001).

For all biomarkers, the highest biomarker levels were observed in the lowest BMI quintiles supporting that the associations were approximately linear across the BMI spectrum. (Supplementary Fig. 1 and Supplementary Fig. 2). Sensitivity analyses restricted to women with BMI <25 kg/m^2^ did not substantively alter the results from any of the combined cohort analyses (Supplementary Fig. 3) supporting that the associations were observed across the BMI spectrum.

### (iii) Association between placental biomarkers and estimated blood volume

Supplementary Table 3 shows the results of univariable models examining the associations between exposures and outcomes in separate regression analyses.

Across cohorts, maternal BMI showed stronger inverse associations than EBV for most biomarkers (Table 3). We found that maternal weight was much better correlated with circulating biomarkers than maternal height. Maternal height showed weak or no correlation with biomarker concentrations (Table 3), suggesting that the inverse associations observed between biomarker concentrations and maternal BMI are not attributable solely to a dilutional effect.

#### (iv) Association between placental biomarkers and BMI genetic risk score

**Figure 2:**
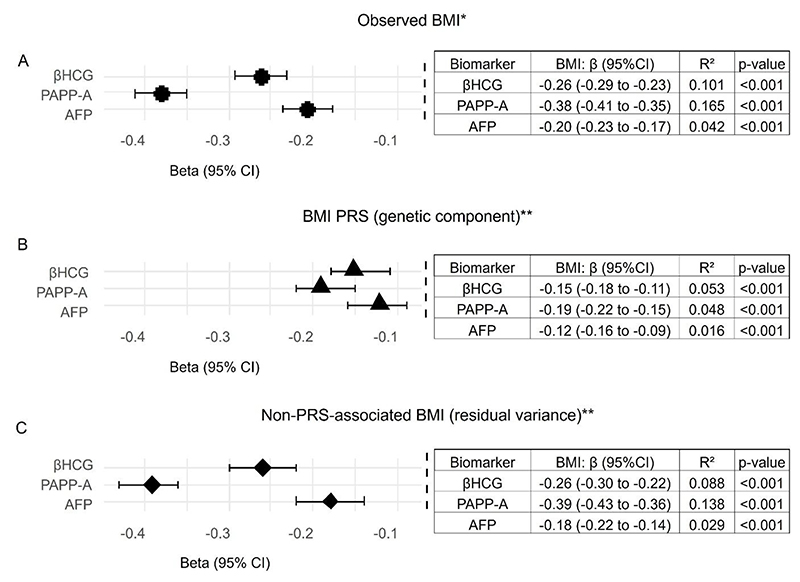
Association between placental biomarkers (gestational age adjusted z scores) and BMI models: observed BMI (A), BMI PRS (B), and non-PRS (C). Left panels show β estimates with 95% CIs, and right panels show the variance explained (R^2^) by each model. GDF15: growth differentiation factor15; βhCG: beta-human chorionic gonadotropin; PAPP-A: pregnancy associated plasma protein A; AFP: alpha-fetoprotein. * Covariates in model: maternal age (standardized), ethnicity and fetal sex. ** Covariates in model: maternal age, PC 1-3 (for ancestry) and fetal sex.

The calculated polygenic risk score (BMI PRS) explained 20.6% of the variability in measured BMI in the POPS cohort. BMI PRS was inversely associated with circulating biomarker concentration for all markers. Observed BMI (phenotype) was more strongly correlated with biomarker concentrations than either the BMI PRS (genetic component) or non-PRS-associated variance in BMI (residual variance). However, residual (non-PRS related) variance was more strongly correlated with biomarker concentrations than PRS-related variance (Fig 2). This suggests that, in the POPS cohort, factors not captured by the PRS may predominantly drive the observed relationships between maternal BMI and circulating biomarkers.

## Discussion

We found inverse associations between early pregnancy BMI and circulating concentrations of the four biomarkers related to placental formation and function. Comparable results were observed across cohorts and remained significant after adjusting for key covariates.

Importantly, our sensitivity analysis restricted to women with BMI <25kg/m2 indicates that our findings do not reflect a specific placentation impairment in women with excess bodyweight. Indeed, the relationship with biomarkers was observed across the full spectrum of BMI, with the highest levels consistently seen among women in the lowest BMI quintile.

Notably, in the recent study of the relationship between maternal BMI and UPVV by Schenkelaars et al., although the authors focus on discussing the effects of obesity, the relationship is most apparent at the lower end of maternal BMI with lean/underweight women having the highest UPVVs [8].

Our sensitivity analysis with respect to estimated blood volume indicates that our findings are not fully explained by a haemodilutional effect. In support of that interpretation, inverse associations of the biomarkers were found more consistently with maternal weight than with maternal height. This was particularly striking in the Cambridge Baby Growth Study, where maternal height was not significantly associated with any of the biomarkers. The obesity polygenic risk score, which captures at least some of the underpinning genetic predisposition to adiposity, also showed a negative association with most biomarkers, providing helpful orthogonal confirmation of the relationship.

We investigated four different biomarkers of early placentation that are detectable in the maternal serum; while the results from each were broadly similar. Three of the markers are secreted by the developing trophoblast (PAPP-A, βhCG, and GDF15), whereas AFP is a fetally-derived protein that passes through the trophoblast to be detectable in the maternal serum [30–33]. While higher gestational-age adjusted concentrations of trophoblast-derived biomarkers in maternal blood might be conceptualised as reflecting either more rapid or more extensive expansion of the materno-fetal interface, the factors influencing AFP concentrations are likely to be more complex. Previous studies suggest that both very high and very low AFP concentrations in maternal blood are associated with suboptimal placentation [34]. This U-shaped relationship is only partially understood, but it has been suggested that low AFP concentrations may reflect impaired transplacental passage of AFP, whereas the extremely high concentrations associated with disease states, e.g. pre-eclampsia, reflect a stage further along the spectrum of impaired placentation where there is breakdown of the maternal-fetal interface [34]. Despite the key difference in origin of AFP and the other three biomarkers, the nature of the relationship with BMI was very similar, suggesting that some common factors are involved; perhaps related to intact development of the materno-fetal exchange surface. It seems apparent that the inverse relationship between placental biomarkers and BMI is not simply driven by obesity but occurs across its full range. It also appears that this relationship is not fully explicable by simple haemodilution [35]. These two findings prompt consideration of other potential explanations.

We propose the hypothesis that, at the earliest stages of placental development, information about maternal caloric storage is sensed by the invading trophoblast and/or the decidua and subsequently influences the establishment of efficient materno-fetal exchange. We assay biomarkers that primarily reflect the developing synctiotrophoblast, although also derive in part from the invading extravillous trophoblast [31,32,36]. As the shift between histiotrophic and haematotrophic nutrition to the developing fetus occurs around 10-13 weeks in human pregnancy, this period may represent a critical window during which maternal nutritional status influences placental formation and function [37]. Although nutritional support of the human conceptus prior to the development of the definitive chorionic villi is poorly understood, there is evidence that synctiotrophoblast do phagocytose glycoproteins from the uterine glands for use in anabolic pathways [37]. The phenomenon of the Arias-Stella reaction, an endocrine-mediated change in the human endometrium in response to the presence of trophoblast, suggests that the placenta does stimulate its own support from maternal resources [38]. Thus, increased concentrations of trophoblast biomarkers in the maternal blood during this crucial period of placental development may reflect increased direct access to maternal resources, in addition to the establishment of a larger or more efficient interface for exchange later in pregnancy.

Regulation of nutrient exchange between the mother and the fetus according to maternal nutrient availability would limit later fetal growth in a calorie-replete environment and promote growth in a marginal environment. Such fluctuations in caloric availability would have occurred frequently throughout the course of human evolution, particularly during the periods of migration which have characterised our species [39].

In mammals there is a clear link between reproductive strategy and nutritional status [40]. It has long been recognised that when maternal energy stores are inadequate, a fall in serum leptin results in reproduction being paused via suppression of ovulation [41]. However, in many species, including humans, pregnancy can still occur when maternal nutrition is marginal [42]. Under these circumstances the fetus will be at risk of poor growth due to lack of substrate. By contrast, a fetus exposed to an excess of maternal glucose and other nutrients is at risk of over-growth and subsequent labour dystocia [43]. The excess of adverse outcomes in babies born both small and large for gestational age, seen consistently in human populations across all global contexts, illustrates the key need to regulate nutritional availability to maintain a normal fetal growth trajectory. However, mechanisms underlying the regulation of nutrient availability to the fetus during pregnancy remain unclear [5,6,44].

We hypothesise that the extent of materno-fetal nutrient exchange is responsive to the ambient caloric status of the mother in the earliest stages of pregnancy. Such responsiveness could be most readily achieved by modulation of the receptivity of the maternal endometrium to vascular invasion by the trophoblast by signals of caloric status of the mother. What might such signals be? Conceivably, macronutrients themselves in the form of circulating levels of glucose and lipids could contribute to such a signal but as these levels are under homeostatic control in the mother their dynamic range may not be optimal to provide adequate information. Perhaps more attractive candidates might be found in one or more nutrient sensitive hormones. Leptin, for example, is an adipocyte-derived hormone the circulating levels of which corelate strongly and positively with the mass of fat stored in adipocytes. Leptin has a well-established role in the central control of the reproductive axis so it is tempting to speculate that it might regulate reproduction at multiple levels. Several hormones are rapidly and highly responsive to the ingestion of macronutrients. These include hormones from the islets of Langerhans such as insulin, pancreatic polypeptide and amylin, and entero-endocrine hormone such as glucagon-like peptide-1 (GLP1), gastric inhibitory polypeptide (GIP) and cholecystokinin (CCK). The receptors for many of these hormones are expressed in the endometrium [45,46] but their effects on endometrial function, including decidualisation, has not been explored in depth.

### Strengths and weaknesses

Data from three well-designed population-based prospective pregnancy cohorts are used in this analysis. The consistency of associations across cohorts differing in sample size, demographic composition, and recruitment periods enhances the generalizability of our results. The large sample size and the use of harmonized analytic approaches enable robust comparisons of the results across the study populations. Additionally, the consistency of results from univariate and adjusted models across cohorts improves the validity of the observed associations. It is also notable that our results are consistent with the studies demonstrating a very similar inverse relationship between maternal BMI and UPVV, an independent and more direct measure of the extent of the Materno-fetal vascular interface.

Limitations of the study include the fact that because maternal energy stores cannot be readily assayed directly in large scale population studies, we have used BMI as a proxy. Maternal nutritional status comprises multiple dimensions, including caloric deficit as well as factors such as insulin resistance and glucose exposure. The limitations of using BMI as a marker of nutritional status are extensively described [1]. Maternal BMI was based on self-reported pre-pregnancy weight in ALSPAC and CBGS and measured at recruitment in POPS. Sensitivity analyses were performed comparing self-reported weight to measured weight in POPS, which were highly correlated, as would be expected from existing literature [47].

EBV was derived from BMI and therefore highly correlated with the exposure, limiting our ability to completely disentangle haemodilution from other BMI-related mechanisms.

Not all biomarkers were measured in every cohort; nevertheless, most were assessed in at least two cohorts, allowing us to verify the robustness of our findings. The placental biomarkers evaluated represent only a small portion of the complex biological processes involved in placental formation and function, and highly dynamic maternal physiology during early pregnancy. Future studies will elucidate the mechanisms linking maternal nutritional status with placentation. The cohorts differ meaningfully in the mean BMI and were collected over a decade apart, likely to reflect changes in population health, environment, and risk distribution. A random-effects IPD-MA was used to account for potential heterogeneity in effect magnitude.

## Conclusion

We propose that the establishment of the early placental footprint may be influenced by maternal nutritional status, in particular the maternal state of caloric storage around the time of conception (proxied by BMI), in a manner that serves to maintain healthy rates of early fetal growth across a wide range of maternal body mass.

## Supplementary Material

Supplementary material

## Figures and Tables

**Table 1:** Baseline characteristics of participants presented as mean (SD) or n (%). Variables are presented with their original units.

Characteristic	N*	ALSPAC	N*	CBGS	N*	POPS
Recruitment years	994	1991-1992	1202	2001-2009	3869	2008-2012
Age (year)	794	27.8 (4.9)	966	33.58 (4.2)	3869	30.0 (5.1)
White ethnicity n(%)	699	684 (97.9)	671	639 (95.2)	3804	3590 (94.4)
Height (cm)	697	164.6 (7.0)	912	166.0 (7.2)	3869	165.2 (6.4)
Weight (Kg)	705	61.4 (10.4)	866	66.3 (13.2)	3869	68.4 (13.4)
BMI (Kg/m^2^)	696	22.7 (3.6)	863	24.1 (4.6)	3869	25.1 (4.7)
EBV-LBB (L)	697	4.2 (0.5)	866	4.4 (0.5)	3869	4.5 (0.5)
Male fetus n(%)	828	425 (51.3)	1202	616 (51.2)	3869	1947 (50.3)

BMI: body mass index; EVB: estimated total blood volume; LBB: Lemmens-Bernstien-Brodsky equation for estimation blood volume;

* Numbers meeting our inclusion criteria- included in the final dataset for analysis.

**Table 2:** Biomarker levels described as mean (SD) and median (IQR) in bold. Variables are presented with their original units.

Characteristic	N*	ALSPAC	N*	CBGS	N*	POPS
GA at sampling (days)	994	72.0(10.9)	924	100.6 (9.2)	3869	88.3 (5.4)
GDF15 (pg/ml)	994	15297.6 (8364.3) **13464.3** **(9957.8-18768.7)**	699	16110.7 (6014.9) **14781** **(11975.9- 19460.3)**		
βhCG (mIU/ml)			910	42134.7 (22307.5) **37400.0** **(27425.0- 52375.1)**	3869	72452.8 (33433.4) **65893.0** **(49775.0- 87944.0)**
PAPP-A (IU/ml)			667	5694.9 (4002.4) **4810.0** **(2745.0- 7720.0)**	3869	4183.5 (3220.1) **3541.0** **(2301.0- 5258.0)**
AFP (IU/ml)			910	23.9 (11.1) **22.3 (17.2- 27.8)**	3869	16.6 (11.0) **14.3 (9.9- 20.5)**

GA: Gestational age; GDF15: growth differentiation factor15; βhCG: beta-human chorionic gonadotropin; pregnancy associated plasma protein A (PAPP-A); AFP: alpha-fetoprotein;

* Numbers meeting our inclusion criteria- included in the final dataset for analysis.

**Table 3:** Results from univariable analysis; placental biomarkers’ association with anthropometrics and EBV across the cohort, Placental biomarkers are used as gestational age adjusted z scores, and exposure are used as standard deviations (SD)s.

Cohort	Biomarker	Exposure	β	R^2^	p-value	AIC
ALSPAC	GDF15	BMI (kg/m^2^)	-0.10	0.010	0.022	1765.7
		EBV (L)	-0.14	0.025	<0.001	1755.9
		Height (cm)	-0.11	0.014	0.002	1763.1
		Weight (kg)	-0.12	0.019	<0.001	1778.3
CBGS	GDF15	BMI (kg/m^2^)	-0.24	0.057	<0.001	1732.5
		EBV (L)	-0.20	0.042	<0.001	1744.3
		Height (cm)	-0.03	0.001	0.457	1843.9
		Weight (kg)	-0.24	0.058	<0.001	1764.3
	PhCG	BMI (kg/m^2^)	-0.22	0.053	<0.001	1403.6
		EBV (L)	-0.17	0.035	<0.001	1413.8
		Height (cm)	0.01	<0.001	0.727	1515.3
		Weight (kg)	-0.20	0.050	<0.001	1441.5
	PAPP-A	BMI (kg/m^2^)	-0.38	0.180	<0.001	1236.8
		EBV (L)	-0.31	0.124	<0.001	1270.2
		Height (cm)	-0.03	0.001	0.389	1386.4
		Weight (kg)	-0.38	0.178	<0.001	1258.4
	AFP	BMI (kg/m^2^)	-0.26	0.060	<0.001	1494.9
		EBV (L)	-0.26	0.074	<0.001	1487.4
		Height (cm)	-0.12	0.013	0.006	1636.2
		Weight (kg)	-0.27	0.079	<0.001	1525.4
POPS	PhCG	BMI (kg/m^2^)	-0.27	0.081	<0.001	10335.9
		EBV (L)	-0.25	0.065	<0.001	10397.8
		Height (cm)	-0.04	0.002	0.004	10652.8
		Weight (kg)	-0.27	0.082	<0.001	10329.5
	PAPP-A	BMI (kg/m^2^)	-0.39	0.16	<0.001	10212.6
		EBV (L)	-0.37	0.142	<0.001	10294.3
		Height (cm)	-0.09	0.008	<0.001	10854.7
		Weight (kg)	-0.41	0.72	<0.001	10156.6
	AFP	BMI (kg/m^2^)	-0.20	0.041	<0.001	10704.5
		EBV (L)	-0.23	0.056	<0.001	10643.4
		Height (cm)	-0.10	0.01	<0.001	10823.4
		Weight (kg)	-0.23	0.055	<0.001	10646.4

BMI: body mass index; EBV: estimated blood volume; AIC: Akaike Information Criterion; GDF15: growth differentiation factor15; βhCG: beta-human chorionic gonadotropin; PAPP-A: pregnancy associated plasma protein A; AFP: alpha-fetoprotein; GA: gestational age.

## Data Availability

The informed consent obtained from ALSPAC, CBGS and POPS participants does not allow the data to be made available through any third party maintained public repository. The studies’ website contains details of all the data that is available through a fully searchable data dictionary and variable search tool.

## References

[1] Sweatt K, Garvey WT, Martins C (2024). Strengths and Limitations of BMI in the Diagnosis of Obesity: What is the Path Forward?. Curr Obes Rep.

[2] Catalano PM, Shankar K (2017). Obesity and pregnancy: mechanisms of short term and long term adverse consequences for mother and child. BMJ.

[3] Poston L, Caleyachetty R, Cnattingius S, Corvalán C, Uauy R, Herring S, Gillman MW (2016). Preconceptional and maternal obesity: epidemiology and health consequences. Lancet Diabetes Endocrinol.

[4] Atta N, Ezeoke A, Petry CJ, Kusinski LC, Meek CL (2024). Associations of High BMI and Excessive Gestational Weight Gain With Pregnancy Outcomes in Women With Type 1 Diabetes: A Systematic Review and Meta-analysis. Diabetes Care.

[5] Dearden L, Ozanne SE (2023). Early life impacts of maternal obesity: a window of opportunity to improve the health of two generations. Philos Trans R Soc Lond B Biol Sci.

[6] Lewandowska M (2021). Maternal Obesity and Risk of Low Birth Weight, Fetal Growth Restriction, and Macrosomia: Multiple Analyses. Nutrients.

[7] Yao R, Park BY, Caughey AB (2017). The effects of maternal obesity on perinatal outcomes among those born small for gestational age*. J Matern Neonatal Med.

[8] Schenkelaars N, Schoenmakers S, Faas MM, Willemsen SP, de Vos ES, Steegers-Theunissen RPM (2025). The maternal body mass index and first-trimester placental (vascular) development. Placenta.

[9] Eskild A, Fedorcsak P, Morkrid L, Tanbo TG (2012). Maternal body mass index and serum concentrations of human chorionic gonadotropin in very early pregnancy. Fertil Steril.

[10] Mejia RB, Cox TW, Nguyen EB, Summers KM, Ten Eyck P, Sparks AE, Van Voorhis BJ (2018). Effect of body weight on early hormone levels in singleton pregnancies resulting in delivery after in vitro fertilization. Fertil Steril.

[11] Petry CJ, Ong KK, Burling KA, Barker P, Goodburn SF, Perry JRB, Acerini CL, Hughes IA, Painter RC, Afink GB, Dunger DB (2018). Associations of vomiting and antiemetic use in pregnancy with levels of circulating GDF15 early in the second trimester: A nested case-control study. Wellcome Open Res.

[12] Drugan A, Dvorin E, Johnson MP, Uhlmann WR, Evans MI (1989). The inadequacy of the current correction for maternal weight in maternal serum alpha-fetoprotein interpretation. Obstet Gynecol.

[13] Huang T, Meschino WS, Okun N, Dennis A, Hoffman B, Lepage N, Rashid S, Aul R, Farrell SA (2013). The impact of maternal weight discrepancies on prenatal screening results for Down syndrome. Prenat Diagn.

[14] Fraser A, Macdonald-wallis C, Tilling K, Boyd A, Golding J, Smith Davey S, Henderson J, Macleod J, Molloy L, Ness A, Ring S (2013). Cohort Profile: The Avon Longitudinal Study of Parents and Children: ALSPAC mothers cohort. Int J Epidemiol.

[15] Boyd A, Golding J, Macleod J, Lawlor DA, Fraser A, Henderson J, Molloy L, Ness A, Ring S, Smith GD (2013). Cohort Profile: The ‘Children of the 90s’—the index offspring of the Avon Longitudinal Study of Parents and Children. Int J Epidemiol.

[16] Prentice P, Acerini CL, Eleftheriou A, Hughes IA, Ong KK, Dunger DB (2015). Cohort Profile: the Cambridge Baby Growth Study (CBGS). Int J Epidemiol.

[17] Pasupathy D, Dacey A, Cook E, Charnock-Jones DS, White IR, Smith GCS (2008). Study protocol. A prospective cohort study of unselected primiparous women: the pregnancy outcome prediction study. BMC Pregnancy Childbirth.

[18] Sovio U, White IR, Dacey A, Pasupathy D, Smith GCS (2015). Screening for fetal growth restriction with universal third trimester ultrasonography in nulliparous women in the Pregnancy Outcome Prediction (POP) study: a prospective cohort study. Lancet (London, England).

[19] Gaccioli F, Lager S, Sovio U, Charnock-jones DS, Smith GCS (2017). The pregnancy outcome prediction (POP) study : Investigating the relationship between serial prenatal ultrasonography, biomarkers, placental phenotype and adverse pregnancy outcomes. Placenta.

[20] Olga L, Sovio U, Wong H, Smith GCS, Aiken CEM (2024). Maternal high body mass index, but not gestational diabetes, is associated with poorer educational attainment in mid-childhood. Am J Obstet Gynecol.

[21] Iles-Caven Y, Northstone K, Golding J (2020). Gestation at completion of prenatal questionnaires in ALSPAC. Wellcome Open Res.

[22] Vricella LK, Louis JM, Chien E, Mercer BM (2015). Blood volume determination in obese and normal weight gravidas: the Hydroxyethyl Starch Method. Am J Obstet Gynecol.

[23] Kennedy H, Haynes SL, Shelton CL (2022). Maternal body weight and estimated circulating blood volume: a review and practical nonlinear approach. Br J Anaesth.

[24] Lemmens HJM, Bernstein DP, Brodsky JB (2006). Estimating blood volume in obese and morbidly obese patients. Obes Surg.

[25] Aguree S, Gernand AD (2019). Plasma volume expansion across healthy pregnancy: a systematic review and meta-analysis of longitudinal studies. BMC Pregnancy Childbirth.

[26] Smit RAJ, Wade KH, Hui Q, Arias JD, Berndt SI, Loos RJF (2025). Polygenic prediction of body mass index and obesity through the life course and across ancestries. Nat Med.

[27] PGS Catalog - The Polygenic Score Catalog.

[28] MacK JA, Sovio U, Day FR, Gaccioli F, Cook E, Bayzid N, Cotic M, Dunton NJ, Madhan G, Motsinger-Reif AA, Perry JRB (2025). Genetic Variants Associated With Preeclampsia and Maternal Serum sFLT1 Levels. Hypertens (Dallas, Tex 1979).

[29] pgsc_calc: a reproducible workflow to calculate polygenic scores — Polygenic Score (PGS) Catalog Calculator documentation.

[30] Turco MY, Gardner L, Kay RG, Hamilton RS, Prater M, Hollinshead MS, McWhinnie A, Esposito L, Fernando R, Skelton H, Reimann F (2018). Trophoblast organoids as a model for maternal-fetal interactions during human placentation. Nature.

[31] D’hauterive SP, Close R, Gridelet V, Mawet M, Nisolle M, Geenen V (2022). Human Chorionic Gonadotropin and Early Embryogenesis: Review. Int J Mol Sci.

[32] Kirkegaard I, Uldbjerg N, Oxvig C (2010). Biology of pregnancy-associated plasma protein-A in relation to prenatal diagnostics: an overview. Acta Obstet Gynecol Scand.

[33] Lafuste P, Robert B, Mondon F, Danan JL, Rossi B, Duc-Goiran P, Mignot TM, Nunez EA, Benassayag C, Ferré F (2002). Alpha-fetoprotein gene expression in early and full-term human trophoblast. Placenta.

[34] Mizejewski GJ (2003). Levels of alpha-fetoprotein during pregnancy and early infancy in normal and disease states. Obstet Gynecol Surv.

[35] Nogues P, Dos Santos E, Couturier-Tarrade A, Berveiller P, Arnould L, Lamy E, Grassin-Delyle S, Vialard F, Dieudonne MN (2021). Maternal Obesity Influences Placental Nutrient Transport, Inflammatory Status, and Morphology in Human Term Placenta. J Clin Endocrinol Metab.

[36] Marjono AB, Brown DA, Horton KE, Wallace EM, Breit SN, Manuelpillai U (2003). Macrophage Inhibitory Cytokine-1 in Gestational Tissues and Maternal Serum in Normal and Pre-eclamptic Pregnancy. Placenta.

[37] Burton GJ, Jauniaux E (2023). The human placenta: new perspectives on its formation and function during early pregnancy. Proceedings Biol Sci.

[38] Arias-Stella J (2002). The Arias-Stella reaction: facts and fancies four decades after. Adv Anat Pathol.

[39] Beyer RM, Krapp M, Eriksson A, Manica A (2021). Climatic windows for human migration out of Africa in the past 300,000 years. Nat Commun.

[40] Schneider JE (2004). Energy balance and reproduction. Physiol Behav.

[41] Moschos S, Chan JL, Mantzoros CS (2002). Leptin and reproduction: A review. Fertil Steril.

[42] Lumey LH (1998). Compensatory placental growth after restricted maternal nutrition in early pregnancy. Placenta.

[43] Gaudet L, Ferraro ZM, Wen SW, Walker M (2014). Maternal obesity and occurrence of fetal macrosomia: a systematic review and meta-analysis. Biomed Res Int.

[44] Smith GCS (2004). First Trimester Origins of Fetal Growth Impairment. Semin Perinatol.

[45] Khan D, Ojo OO, Woodward ORM, Lewis JE, Sridhar A, Gribble FM, Reimann F, Flatt PR, Moffett RC (2022). Evidence for Involvement of GIP and GLP-1 Receptors and the Gut-Gonadal Axis in Regulating Female Reproductive Function in Mice. Biomol.

[46] Sola-Leyva A, Pathare ADS, Apostolov A, Aleksejeva E, Kask K, Tammiste T, Ruiz-Durán S, Risal S, Acharya G, Salumets A (2025). The hidden impact of GLP-1 receptor agonists on endometrial receptivity and implantation. Acta Obstet Gynecol Scand.

[47] Natamba BK, Sanchez SE, Gelaye B, Williams MA (2016). Concordance between self-reported pre-pregnancy body mass index (BMI) and BMI measured at the first prenatal study contact. BMC Pregnancy Childbirth.

