# Supplementary material for: The inverse association between circulatory placental biomarkers in early pregnancy and maternal body mass index
Source: Placenta. Author manuscript; Available in PMC 2026 Mar 16. (PMC7618890; doi:10.1016/j.placenta.2026.03.004)
Supplement: Supplementary material [file EMS212787-supplement-Supplementary_material.pdf]

Supplementary material

Supplementary Table 1. Description of biomarker assays in cohorts included in this study.

Cohort	ALSPAC	CBGS	POPS
Biomarkers measured:	GDF15	GDF15, HCG, PAPP-A and AFP	HCG, PAPP-A and AFP
Samples	Serum	Serum	Serum
Assays	GDF15 concentrations were measure in serum samples using a three-step plate ELISA (Ansh AL-1014-r) which was validated to be able to recognize H- and D-containing variants at position 202 (position six of the mature peptide) of GDF15 with comparable affinity.	GDF15 concentrations were measure using a three-step plate ELISA (Ansh AL-1014-r) which was validated to be able to recognize H- and D-containing variants at position 202 (position six of the mature peptide) of GDF15 with comparable affinity.<br>PAPP-A was measured by time-resolved fluoroimmunoassay (AutoDELFIA; Perkin Elmer, Seer Green, UK).<br>AFP and HCG were measured as prenatal screening assays using routine AutoDELFIA time-resolved fluoroimmunoassays (PerkinElmer Life Sciences, Wallac Oy, Turku).	Maternal serum concentrations of HCG, PAPP-A and AFP, were measured using Roche Elecsys assays on the electrochemiluminescence immunoassay platform, Cobas e411 (Roche Diagnostics).

GDF15: growth differentiation factor15; βhCG: beta-human chorionic gonadotropin; PAPP-A: pregnancy associated plasma protein A; AFP: alpha-fetoprotein.

Supplementary Table 2. Analysis plan

Study plan: The association between maternal BMI in early pregnancy on placental formation and function

Background and rational

Preliminary evidence suggests a link between maternal nutritional status during the periconceptional period and the extent of placental invasion. We hypothesise that this may represent a beneficial evolutionary mechanism designed to optimise reproductive success under a variety of environmental nutritional challenges. In mammals there is a clear link between reproductive strategy and nutritional status. It has long been recognised that when energy available to the mother is inadequate to support adipose triglyceride stores the fall in serum leptin results in reproduction being paused via suppression of ovulation to promote survival. However, in many species, pregnancy can occur when maternal nutrition is marginal. Under these circumstances the fetus will be at risk of poor growth due to lack of substrate. By contrast, a fetus exposed to an excess of maternal glucose and other nutrients is at risk of over-growth and subsequent labour dystocia. The excess of adverse outcomes in babies born both small and large for gestational age, seen consistently in human populations across all global contexts, illustrates the key need to regulate nutritional availability to maintain a normal fetal growth trajectory. However, mechanisms underlying the regulation of nutrient availability to the fetus during pregnancy are not known. We propose a simple theory, in which the extent of early trophoblast invasion is mediated by maternal nutritional status. Thus, a conceptus in a nutritionally-poor environment would establish more extensive trophoblast invasion of the decidua, leading to a greater placental footprint and increased capacity for nutrient transfer. By contrast, trophoblast invasion in the over-nourished mother may be less extensive, hence limiting fetal nutrient exposure and thus growth. Maternal energy stores cannot be readily assayed directly in large scale population studies, but we intend to use body mass index (BMI) as a proxy. The limitations of using BMI as a marker of nutritional status are extensively described elsewhere (1), but it serves as a pragmatic and clinically-relevant proxy for this investigation. We will also examine the correlation with maternal blood volume (to take into account the volume of distribution of placentally-secreted hormones).

Placental invasion will be proxied by measuring circulating hormone levels released from the invading trophoblast, which will be expressed as gestational age-adjusted z-scores to account for rapidly increasing levels as gestation progresses.

We hypothesize that maternal body mass index will be associated with maternal serum concentrations of placentally-derived hormones in early pregnancy (~8-16 wks gestation).

Main variables

We intend to use following variables:

Exposure variables: Maternal BMI (in kg/m^2) as a numeric variable. BMI will be calculated using measured or self-reported height (m) and weight (kg) at early pregnancy. Height and weight will also be assessed as independent exposure variables and further converted to estimate blood volume (EBV).

Two equations are commonly used to estimate blood volume:

Nadler (2): $EBV = (0.3561 \times H^3) + (0.03308 \times W) + 0.1833$

Lemmens-Bernstien-Brodsky (3): $EBV = 70/[\sqrt{BMI} \div 22])$

We will use the LBB method, as other studies suggest it shows better fit at extremes of BMI range, but perform a sensitivity analysis using the Nadler method (4).

Pregnancy-specific methods of calculating EBV are available, but these have been derived for use in the third trimester. In a meta-analysis, Aguree et al. 2019, concluded that plasma volume increases by only 6% in the first trimester v. 42-48% in the third trimester (5), therefore we will use non-pregnancy equations in our estimates.

Outcome variables: We will include following biomarkers of placentation adjusted for gestational age at sampling (in days).

Table 1. Description of the biomarkers of placental invasion and their function.

Biomarkers	Description
βHCG (mIU/ml)	Human chorionic gonadotropin is a heterodimeric hormone, with an alpha subunit identical to that of pituitary-secreted gonadotrophic hormones. The beta subunit (βHCG) is a unique bioactive peptide that promotes progesterone production by corpus luteal cells and supports the maintenance of the endometrial lining. It is secreted only by syncytiotrophoblasts starting 6–7 days post-fertilization, and hence is used in pregnancy testing. βHCG peaks at around 10–12 weeks of gestation.<br><br>βHCG reflects total syncytiotrophoblast mass, and hence rise in βHCG is used primarily as a biomarker of pregnancy viability, with a ≥60% rise in 48hrs indicating sufficient increase in trophoblast mass during early pregnancy. We will use βHCG as a surrogate marker of total syncytiotrophoblast mass, normalized to gestation.

PAPP-A (IU/ml)	Pregnancy-associated plasma protein-A (PAPP-A) is widely secreted by tissues within the reproductive tract (e.g. granulosa cells) and elsewhere (e.g. adipose tissues). During early pregnancy, circulating levels increase rapidly due to secretion from the developing syncytiotrophoblast. This glycoprotein plays a critical role in trophoblast invasion, implantation, and fetal growth by regulating the bioavailability of insulin-like growth factors (IGFs). Its production begins early in pregnancy, around the time of implantation.<br><br>PAPP-A is a metalloprotease that specifically cleaves IGF binding proteins, making IGF1 available as a growth factor. PAPP-A is the only known protein whose concentration in early pregnancy is sufficiently associated with fetal growth restriction to use as a single biomarker. We will use PAPP-A as a surrogate marker of placental growth.
GDF15 (pg/ml)	During pregnancy, Growth differentiation factor 15 (GDF15) is produced by both syncytiotrophoblasts and extravillous trophoblasts. Syncytiotrophoblast secretes GDF15 into maternal circulation where it exerts its effects, entirely mediated by its receptor GFRAL-Ret which is exclusively located in two nuclei of the hindbrain. These responses include changes in appetite, aversions to certain foods, and activation of the hypothalamic pituitary adrenal axis. GDF15 production begins early in pregnancy, gradually rising until the third trimester. Like βHCG, we will use GDF-15 levels as a surrogate marker for trophoblast expansion.
AFP (IU/ml)	During pregnancy, alpha-fetoprotein (AFP) is primarily produced by the fetal liver and yolk sac, with smaller contributions from the gastrointestinal tract. AFP enters the maternal circulation through diffusion across the placental membranes and reflects both fetal production and placental transfer efficiency. Maternal serum AFP levels rise steadily throughout gestation, peaking around 32 weeks. Because AFP concentrations are influenced by placental permeability and surface area, we will use maternal AFP levels as an indirect marker of placental function and materno-fetal exchange.

Covariates: Maternal characteristics, that might interfere the relationship between the exposures of interest and placental development will be included in the multivariable analysis. In addition to the gestational age, we aim to include following covariates in the analysis:

Table 2. Description of the covariates that will be included in our analysis.

Maternal Age (numeric)	Maternal age can affect placental development, primarily by altering angioger[illegible] trophoblast invasion. For example in women aged 35–45 lower PlGF and i[illegible] sFlt-1, resulting in decreased placental blood flow.
Ethnicity (white /non-white)	It is plausible that systematic differences exist between establishment of pla[illegible] in women of different ethnicities.
Fetal sex (m/f)	Systematic differences exist in the secretome of early trophoblasts with [illegible] karyotype

Datasets: POPS: The Pregnancy Outcome Prediction Study (POPS) recruited unselected nulliparous women with singleton pregnancies at The Rosie Hospital, Cambridge, England, between January 2008 and July 2012 (n = 4,212). The database contains information from four study visits at 12 weeks (anthropometry and phlebotomy), and at 20, 28, and 36 weeks (fetal biometry, liquor volume, phlebotomy, uterine and umbilical Doppler).

CBGS: The Cambridge Baby Growth Study (CBGS) recruited women aged ≥16 years, irrespective of parity, in early pregnancy (~12 weeks) at the Rosie Maternity Hospital, Cambridge, UK, between April 2001 - March 2009.

ALSPAC: ALSPAC (The Avon Longitudinal Study of Parents and Children, formerly the Avon Longitudinal Study of Pregnancy and Childhood) is a birth cohort that recruited women with expected delivery dates between April 1991 to December 1992 (ALSPAC G0), residing in and around Bristol, UK. Access to the ALSPAC dataset was given after approval of proposal # B4963.

Analytic sample: We will include all pregnancies that resulted in a liveborn baby at >24 weeks gestation with no congenital anomaly that might have influenced early placentation (e.g. trisomies). We will exclude pregnancies where (i) none of the outcome variables have been assayed in the blood sample taken at early pregnancy and (ii) no information on early pregnancy height and weight is available.

Analytic strategy: Our analysis will consist of the following steps:

Step 1: Data visualization: We will visualize the relationships between each of our exposures and outcomes of interest (primarily using scatterplots and lines of best fit, both linear and non-linear). We will provide correlation coefficients (r) and p values where appropriate.

Step 2: Univariate models: We will perform univariate analysis examining the association between our exposures and outcome variables.

We will perform testing to determine whether maternal BMI or EBV as an exposure fit our outcomes better. To determine which exposure (maternal BMI or EBV) explains more of the variability of the outcomes, we will fit both models and may compare their Akaike Information Criterion (AIC) values: ΔAIC=AICmodel1−AICmodel2. The model with a smaller AIC value will indicate better fitting. We will also compare the strength of the associations (e.g. B coefficients, R^2 and p-value) in both models to see which one is better where there is biological plausibility.

Step 3: Multivariable models: We will perform multivariable analyses examining the effect of our exposures on our outcomes with the addition of the specified co-variates, selecting these for retention in the final models where there is high biological plausibility or improved model fit (assessed by AIC and/or metrics listed in the table below) when they are included.

For biomarkers measured in more than one cohort, we will pool the raw data from each cohort to jointly estimate associations while accounting for cohort-specific variability using random effects models in individual participant data meta-analyses (IPD-MA).

Polygenic risk scores (PRS) for BMI will be generated using data from the POPS cohort and will be used as a sensitivity analysis to assess the relation with the biomarkers.

Statistical Methods: Our main analysis methods are as follows

Table 3: analysis methods

**Methods**	**Outcomes**
Descriptive analyses	Descriptive comparisons between groups<br>For numerical normally-distributed variables group differences will be presented using presented in mean± standard deviation (SD); for non-normal distributed variables medians and interquartile range will be used. For categorical variables (N and %).
Correlation analysis	Correlation coefficients and p values (Pearson's for normal distribution and Spearman's for non-normal distribution)
Association analyses	Where appropriate t-test, Mann Whitney U test,chi-squared test;
Linear (and non-linear) regression models	B coefficients, standard errors, goodness of fit-statistics (e.g. R2, R2 Change, AIC and BIC) and p values. For any selection of covariates, we may use nested model comparisons (e.g. stepwise), cross-validation, or regularisation

*All analyses will be conducted using R version 4.3.3. Alternative software will be stated where used.

No imputation will be performed and cases for which no assays of hormone concentrations have been performed will be assumed to be missing at random, where the entire cohort has previously undergone analysis. Data outliers will be verified for accuracy, and unexplained extreme values will be excluded from analysis.

Power Calculations: The number of samples tested for placental biomarkers at POPs visit 1 is reported as n=4078 (7). Assuming this sample size (n=4078), an alpha of 0.05 (two-tailed), and a power ≥ 80%, the smallest detectable effect size was calculated using package "pwr" in R. In a linear regression model with three predictors and a sample size of n = 4078 (and above-mentioned parameters), an effect size as small as 0.27% can be detected (R^2 = 0.0027 Cohen's f^2 = 0.0027). With one predictor it was R^2 = 0.0019 (Cohen's f^2 = 0.0019). Increasing the number of predictors to 10, the smallest detectable effect size for the n=4078 was R^2=0.0040 (f^2=0.0040), and at predictors=20 the effect size is f^2=0.0052

Supplementary Table 3. Correlations (r) between BMI, EBV, height, weight and placental biomarkers in ALSPAC (A), CBGS (B) and POPS (C) cohorts.

**A) ALSPAC**	BMI	EBV	Height	Weight	GDF15			
BMI	1	0.62	-0.1	0.87	-0.1			
EBV	0.62	1	0.71	0.92	-0.16			
Height	-0.1	0.71	1	0.39	-0.1			
Weight	0.87	0.92	0.39	1	-0.14			
GDF15	-0.09	-0.16	-0.12	-0.14	1			
**B) CBGS**	BMI	EBV	Height	Weight	β- hCG	PAPP_A	AFP	GDF15
BMI	1	0.68	-0.10	0.90	-0.23	-0.42	-0.25	-0.24
EBV	0.68	1	0.65	0.93	-0.19	-0.35	-0.27	-0.20
Height	-0.10	0.65	1	0.34	-0.01	-0.04	-0.13	-0.03
Weight	0.90	0.93	0.34	1	-0.22	-0.42	-0.28	-0.24
β- hCG	-0.23	-0.19	-0.01	-0.22	1	0.37	0.19	0.53
PAPP-A	-0.42	-0.35	-0.04	-0.42	0.37	1	0.22	0.36
AFP	-0.25	-0.27	-0.13	-0.28	0.19	0.22	1	0.21
GDF15	-0.24	-0.20	-0.03	-0.24	0.53	0.36	0.21	1
**C) POPS**	BMI	EBV	Height	Weight	β- hCG	PAPP_A	AFP	
BMI	1.00	0.73	-0.08	0.92	-0.28	-0.40	-0.20	
EBV	0.73	1.00	0.62	0.94	-0.26	-0.38	-0.24	
Height	-0.08	0.62	1.00	0.32	-0.05	-0.09	-0.11	
Weight	0.92	0.94	0.32	1.00	-0.29	-0.41	-0.24	
β- hCG	-0.28	-0.26	-0.05	-0.29	1.00	0.32	0.05	
PAPP-A	-0.40	-0.38	-0.09	-0.41	0.32	1.00	0.09	
AFP	-0.20	-0.24	-0.11	-0.24	0.05	0.09	1.00	

BMI: body mass index; EBV: estimated blood volume; GDF15: growth differentiation factor15; βhCG: beta-human chorionic gonadotropin; PAPP-A: pregnancy associated plasma protein A; AFP: alpha-fetoprotein.

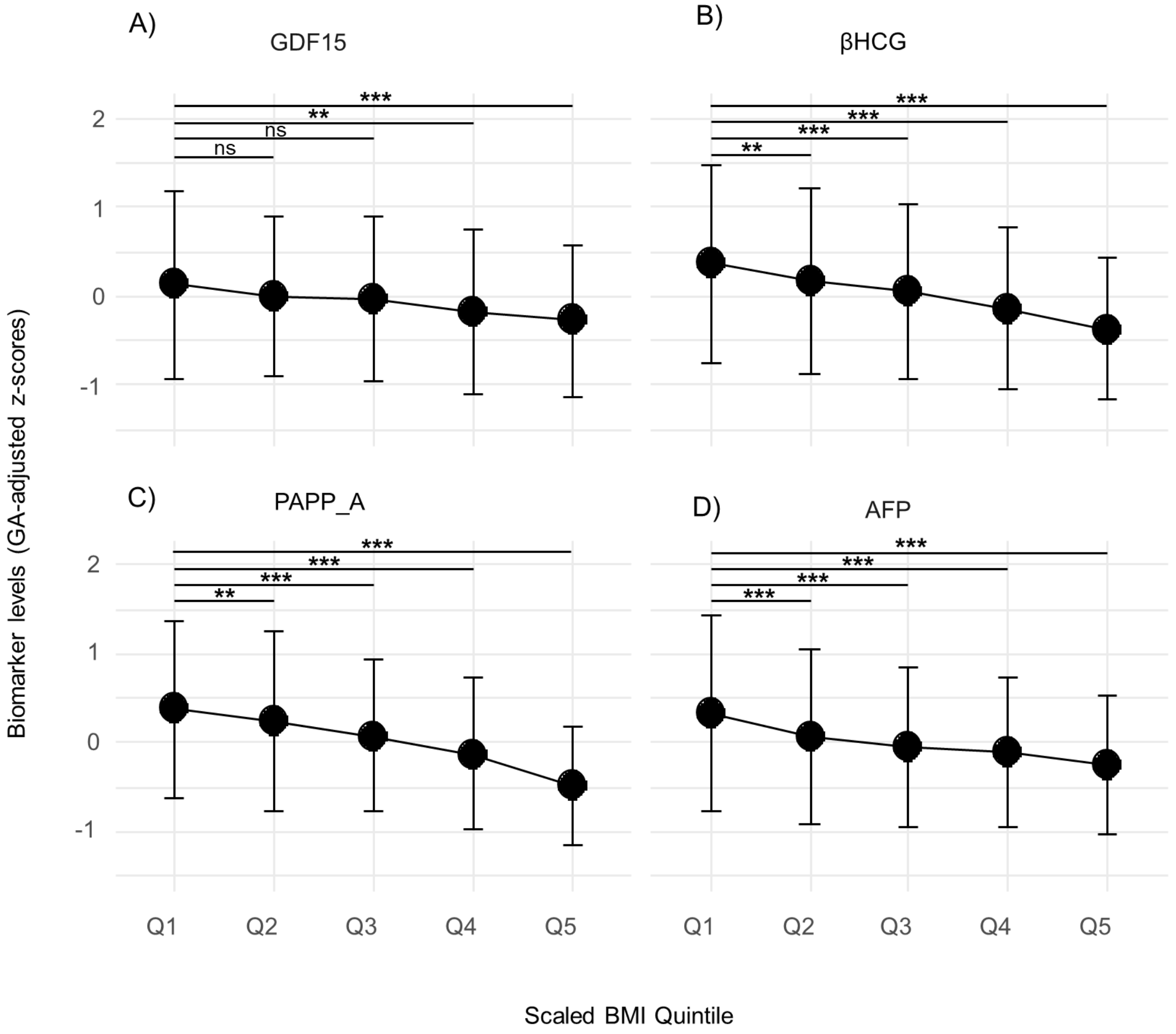

Supplementary Figure 1. Mean ± 2SD of biomarker levels across BMI quintiles (Q1–Q5). Pairwise comparisons were made between Q1 and each of the other quintiles using t-tests, with Bonferroni adjustment for multiple testing. GDF15: growth differentiation factor15; βhCG: beta-human chorionic gonadotropin; PAPP-A: pregnancy associated plasma protein A; AFP: alpha-fetoprotein.

Adjusted P-value>0.05 (ns), <0.05 (*), <0.01 (**), <0.001 (***).

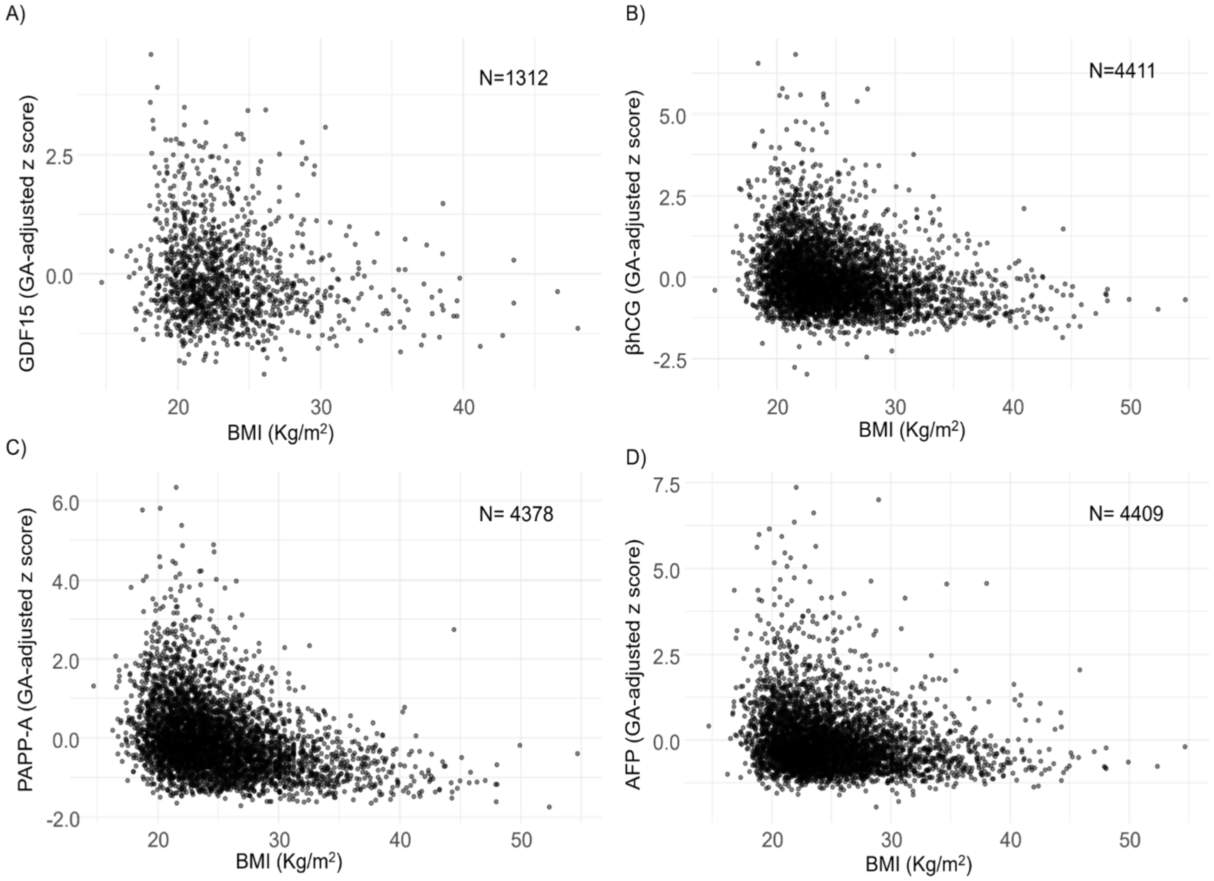

Supplementary Figure 2. Scatter plots of the placental biomarkers against BMI in pooled dataset.

BMI; body mass index; GDF15: growth differentiation factor15; βhCG: beta-human chorionic gonadotropin; AFP: alpha feto protein;

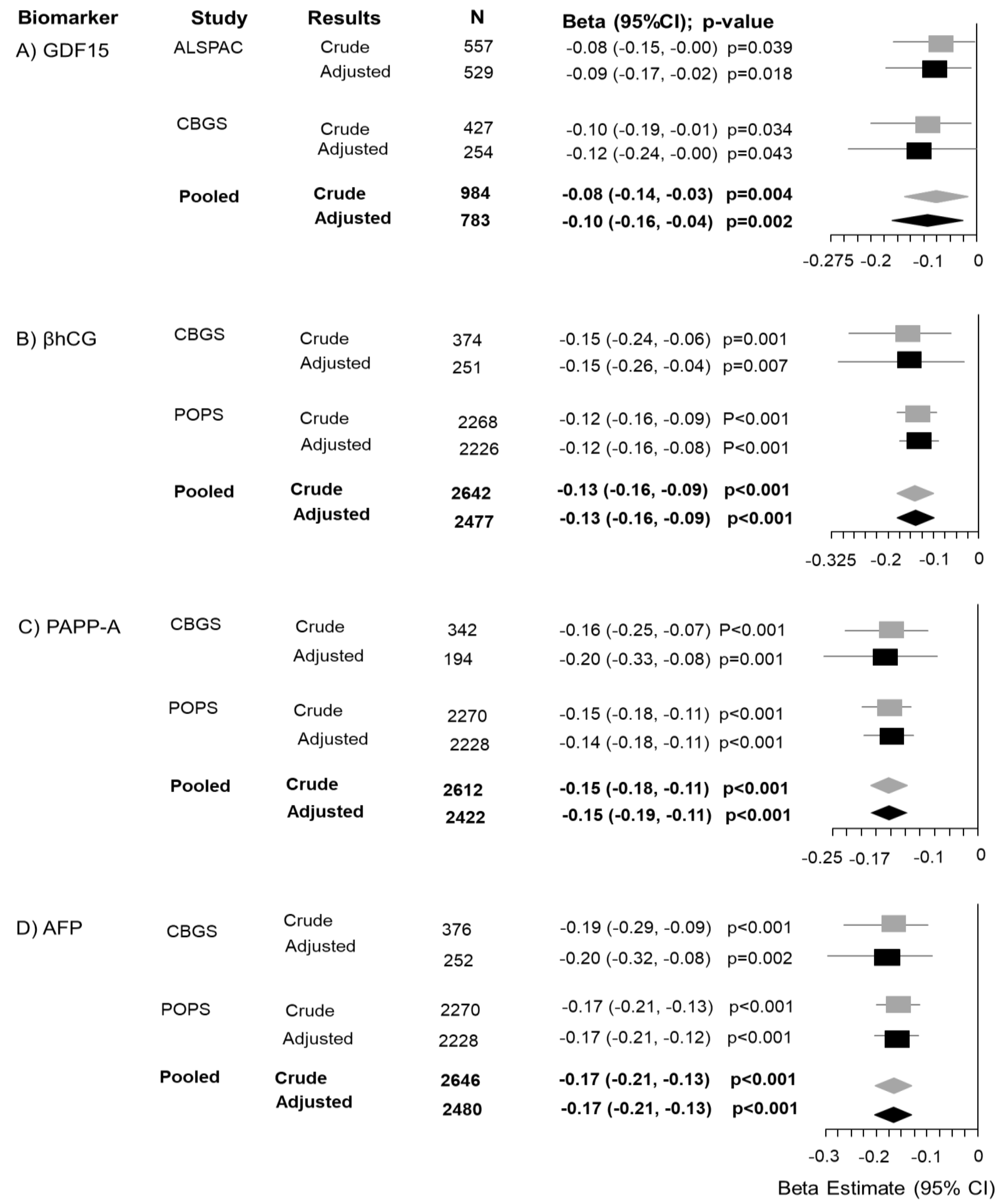

Supplementary Figure 3. Results from individual participant data meta-analysis (IPD-MA) in women with BMI <25. The forest plots show cohort-specific and pooled associations between maternal BMI and placental biomarkers.
GDF15: growth differentiation factor15; βhCG: beta-human chorionic gonadotropin; PAPP-A: pregnancy associated plasma protein A; AFP: alpha-fetoprotein.
*Adjustments: maternal age (standardized), ethnicity and fetal sex.
